# Factors associated with death in severe patients with COVID-19/diabetes mellitus in Brazil

**DOI:** 10.1590/0034-7167-2024-0361

**Published:** 2025-12-15

**Authors:** Isabella Rodrigues da Silva Batista Lima, Andressa Jhulier Faiola Oliveira, Lívia Maia Pascoal, Floriacy Stabnow Santos, Leonardo Hunaldo dos Santos, Marcelino Santos, Janaina Miranda Bezerra, Ana Cristina Pereira de Jesus Costa

**Affiliations:** I Universidade Federal do Maranhão. Imperatriz, Maranhão, Brazil

**Keywords:** Diabetes *Mellitus*, COVID-19, Severe Acute Respiratory Syndrome, Risk Factors, Mortality., Diabetes *Mellitus*, COVID-19, Síndrome Respiratorio Agudo Grave, Factores de Riesgo, Mortalidad.

## Abstract

**Objectives:**

to analyze factors associated with death in severe COVID-19/diabetes *mellitus* patients in Brazil.

**Methods:**

a cross-sectional study, carried out with notifications of severe respiratory diseases due to COVID-19 registered in the Brazilian Healthcare system database, between 2020 and 2022, Brazil. To analyze associated factors, univariate and multivariate logistic regression models were used.

**Results:**

males, aged 19 to 59 years, older adults, non-white race/skin color, low education level, nosocomial cases, Intensive Care Unit admission, ventilatory support, chronic cardiovascular and renal diseases, asthma, chronic lung diseases, immunosuppression, clinical manifestations, serological tests, and imaging were associated with a higher risk of death. Variables such as rural area, cough, sore throat, and antigen test not performed were protective factors.

**Conclusions:**

these data reinforce the need for strategies based on social determinants of health to expand access, clinical management, and reduce mortality in these patients.

## RESUMEN

**Objetivos:**

analizar los factores asociados con la muerte en pacientes graves con COVID-19/diabetes *mellitus* en Brasil.

**Métodos:**

estudio transversal, realizado con las notificaciones de enfermedades respiratorias graves por COVID-19 registradas en la base de datos del Sistema Único de Salud, entre 2020 y 2022, Brasil. Para analizar los factores asociados, se utilizaron modelos de regresión logística univariados y multivariados.

**Resultados:**

los varones de 19 a 59 años, adultos mayores, de raza/color de piel no blanco, con bajo nivel educativo, casos nosocomiales, ingreso en unidad de cuidados intensivos, soporte ventilatorio, enfermedades cardiovasculares y renales crónicas, asma, enfermedades pulmonares crónicas, inmunosupresión, manifestaciones clínicas, pruebas serológicas e imagenología se asociaron con un mayor riesgo de muerte. Las variables zona rural, tos, dolor de garganta y no realización de prueba de antígenos fueron factores protectores.

**Conclusiones:**

estos datos refuerzan la necesidad de estrategias basadas en los determinantes sociales de la salud para ampliar el acceso, la atención clínica y reducir la mortalidad en estos pacientes.

## INTRODUCTION

COVID-19, a disease caused by SARS-CoV-2 infection, is a severe acute respiratory disease with a high potential for transmissibility. In addition to respiratory involvement, COVID-19 can cause multisystemic and prolonged disease involvement in some individuals^(1,2)^. According to the report by the Pan American Health Organization, during the 30^th^ Pan American Sanitary Conference, which took place in September 2022, the Americas region was responsible for reporting 30% and 44% of cases and deaths from COVID-19 in the world, respectively. Among the countries of the Americas, the United States of America and Brazil stood out among the ten countries with the highest number of accumulated cases on a global scale^(3)^.

Symptoms associated with COVID-19 infection can range from mild to severe, including severe acute respiratory syndrome (SARS), which is characterized by peripheral oxygen saturation (SpO_2_) <95%, hypotension, respiratory distress, and worsening clinical conditions of pre-existing diseases^(4)^. Since the first reports, many severe and fatal cases of COVID-19 have occurred in older patients or people with comorbidities, especially those with diabetes *mellitus* (DM), hypertension, cerebrovascular diseases, chronic renal, and pulmonary diseases^(5)^. It is also worth noting that people with DM have a 2.3 times greater risk of developing more severe forms of the disease, in addition to having a 2.5 times greater risk of mortality related to COVID-19^(6)^.

DM is emerging as a growing public health challenge, as projected by the International Diabetes Federation. Around 537 million adults aged 20 to 79 worldwide have this condition, accounting for 10.5% of the global population. Estimates suggest a significant increase of 46% in the number of people living with DM by 2045. Furthermore, this increase, during the same period, will be more pronounced in nations characterized by low and middle income, reflecting population growth in these regions^(7)^.

The relationship between DM and other infectious diseases can be attributed to the difficulty that people with DM have in mounting an effective immune response against infections, which increases their vulnerability and predisposition to developing active disease. Because of this condition, people with diabetes are classified as a high-risk group for infectious diseases, including SARS-CoV-2 infection^(8)^; however, there are still gaps regarding this interrelationship. The literature indicates that there is a bidirectional link between the pathophysiology of DM and the COVID-19 virus so that DM contributes to the development of complications when patients are infected with the coronavirus^(9)^. Associated with this complex relationship, it is possible to consider that DM contributes to unfavorable clinical results, increasing the risk of death^(10)^. Moreover, hyperglycemia at hospital admission was identified as an indicator of all-cause mortality in patients hospitalized with COVID-19 who were not in critical condition, regardless of the previous history of diabetes^(11)^.

Regarding complications, a study conducted at a referral hospital for severe cases of COVID-19 in the city of Jakarta, Indonesia, revealed that DM increased the risk of intensive care treatment by 2.57 times compared to those who did not have DM^(10)^. Another study showed that, in the group of people with DM, complications were observed more frequently than in the group without DM, and the most prevalent were acute respiratory distress, acute cardiac injury and acute kidney injury^(12)^.

In addition to clinical factors, socioeconomic characteristics such as low education, non-white race/color and limited access to healthcare services are associated with an increased risk of mortality in this population group^(13)^. The interrelationship between social and biological factors highlights the importance of addressing health vulnerability from a multidimensional perspective, as outlined by the World Health Organization’s social determinants of health (SDH) models. These models highlight how structural inequalities shape clinical outcomes and influence health determinants in complex and interdependent ways^(14)^.

Considering the potential occurrence of disabling complications of the disease itself, in addition to future infections that may arise and become potentially fatal, DM is an essential component of the Strategic Action Plan for Confronting Chronic Diseases and Non-Communicable Diseases (NCDs) in Brazil, 2021-2030^(15)^. As a priority for health promotion, DM is also aligned with the global commitments advocated by the Sustainable Development Goals (SDGs) of the 2030 Agenda, in particular SDG 3 (“Good Health and Well-being”). Target 3.4 aims to reduce premature mortality from chronic non-communicable diseases by one third by 2030, reinforcing the need to address the complexities of chronic conditions such as DM^(16)^.

Although evidence shows that people with DM are at risk of contracting COVID-19, it is important to understand the factors associated with death. This understanding allows us to develop strategies to prevent serious outcomes, facilitating the implementation of targeted actions for prevention and adequate care for these individuals. Given the significant representation of this group in the population susceptible to serious complications from the disease, the intersection between COVID-19 and DM not only highlights public health challenges, but also sheds light on fundamental issues related to global well-being.

## OBJECTIVES

To analyze factors associated with deaths in severe patients with COVID-19/DM in Brazil.

## METHODS

### Ethical aspects

Patient consent was waived, as the research is based solely on secondary data available on OpenDATASUS. This government platform offers comprehensive access to health data in Brazil in a public and anonymized format, without presenting risks to participants.

### Study design, period and place

This is a cross-sectional study, conducted using the STrengthening the Reporting of OBservational studies in Epidemiology instrument, carried out with reported data on SARS in the period from February 25, 2020 to September 30, 2022, in Brazil, a country located in South America, which is home to an estimated population of 213,759,869 inhabitants, distributed across 27 Federative Units and five macro-regions (South, Southeast, Central-West, North and Northeast)^(17)^. It is worth noting that the start period corresponds to the notification of the first case of COVID-19 registered in the country.

### Population or sample; inclusion and exclusion criteria

The population consisted of all records of SARS due to COVID-19 associated with DM, reported in the OpenDATASUS database during the study period. Reports in which the person had DM (regardless of type 1 or 2 classification) were included, and reports in which patients did not test positive for COVID-19 were excluded. Cases with confirmation of the SARS-CoV-2 virus in the notification form were considered positive for COVID-19, through tests such as molecular biology real-time polymerase chain reaction (RT-PCR), indicative findings on X-ray and chest tomography, in addition to rapid tests for SARS-CoV-2 positive for IgM and/or IgG antibodies. Incomplete information in the notification form that did not specify whether patients had DM, the outcome of the case, and reports of deaths outside the study period were disregarded.

### Study protocol

In this study, the SDH were adopted as a theoretical model due to their multidimensional approach, expressing two interconnected levels of determination: the structural ones, linked to sociodemographic conditions, and the intermediate ones, related to clinical-biological aspects, also incorporating access to and quality of healthcare services, which allows us to analyze how such factors influence deaths in severe patients with SARS due to COVID-19 and DM in Brazil.

In this study, these categories were operationalized based on sociodemographic and clinical-epidemiological variables extracted from the analyzed database. Structural determinants (sex, age, race/color, education, area of residence, and gestational status) influence intermediate determinants (comorbidities and clinical signs and symptoms), which, in turn, interact dynamically with factors related to access and healthcare (epidemiological history and type of care received). The effectiveness of care is measured through diagnostic tests and clinical criteria applied, which, together, contribute to the outcome of death due to SARS-COVID-19. To structure the analysis of these relationships, this study adapted the theoretical model of SDH proposed by Solar and Irwin (2010)^(14)^.

Data collection took place in October 2022, using information from the OpenDATASUS database (opendatasus.saude.gov.br/dataset/bd-srag-2021), linked to the Ministry of Health. Clinical and sociodemographic variables with an outcome of death from SARS due to COVID-19 were selected, namely: sex (male and female); age (children: 0 to 11 years; adolescents: 12 to 18 years; adults 19 to 59 years; and older adults: over 60 years); pregnant woman (1^st^ trimester, 2^nd^ trimester, 3^rd^ trimester, gestational age unknown, no and not applicable); race/color (white, black, yellow, brown and indigenous); education (no education, elementary school 1^st^ cycle: 1^st^ to 5^th^ grade; elementary school 2^nd^ cycle: 6^th^ to 9^th^ grade; high school: 1^st^ to 3^rd^ grade; and higher education); area (urban, rural and peri-urban); risk factors/comorbidities (DM and chronic cardiovascular disease, chronic hematologic disease, puerperal, Down syndrome, chronic neurological disease, chronic lung disease, immunodeficiency or immunosuppression, chronic kidney disease, chronic liver disease, asthma and obesity); signs and symptoms (fever, cough, sore throat, dyspnea, respiratory distress, oxygen saturation, diarrhea, vomiting, abdominal pain, fatigue, loss of smell and loss of taste), care data (outbreak of influenza-like syndrome, nosocomial case, work with poultry and swine, flu vaccine, antiviral for flu, hospital/hospitalization, Intensive Care Unit (ICU) and ventilatory support); chest X-ray (normal, interstitial infiltrate, consolidation, mixed, other and not performed); type of sample collected for testing (nasopharyngeal secretion, bronchoalveolar lavage and *post-mortem* tissue); PCR (detectable, not detectable, inconclusive, not performed and awaiting result); tomography appearance (typical COVID-19, indeterminate COVID-19, atypical COVID-19, negative for pneumonia and not performed); types of antigenic test (immunofluorescence and rapid antigenic test); antigenic test result (positive, negative, inconclusive, not performed and awaiting result); IgM serological test (positive, negative, inconclusive, not performed and awaiting result); and closure criteria (laboratory, link/epidemiological and clinical).

### Analysis of results and statistics

After identifying possible errors and inconsistencies, a descriptive analysis was conducted, in which absolute and relative frequencies were presented for sociodemographic and clinical-epidemiological variables. To assess possible risk factors for death from SARS due to COVID-19 among sociodemographic and clinical-epidemiological variables, simple and multiple logistic regression models were used^(18)^, considering the binary response (death: yes or no). In the selection of the main risk factors, univariate logistic analysis (not adjusted) was initially performed, with selection criterion p<0.20.

Multicollinearity of independent variables was tested based on the variance inflation factor, calculated by multiple linear regression, whose cut-off point was variance inflation factor > 10, with no multicollinearity observed in any of the cases. The presence of autocorrelation was also tested using the Durbin-Watson test for both models, indicating homogeneity of variance of residuals. Selected variables were then subjected to multivariate (adjusted) logistic regression using the backward stepwise selection method. The best model was selected based on the lowest Akaike Information Criterion value. Odds Ratios were also calculated, accompanied by 95% Confidence Intervals. All tests were performed using the IBM Statistical Package for the Social Sciences version 24.0, with a significance level of 5%.

## RESULTS

From 2020 to 2022, 3,381,018 cases of SARS were reported in Brazil. After applying the inclusion and exclusion criteria, 484,139 cases had COVID-19/DM, of which 193,591 resulted in death. Table 1 shows the univariate and multivariate logistic regression analysis of sociodemographic and clinical characteristics of deaths in severe patients with COVID-19/DM in Brazil from 2020 to 2022.

**Table 1:** Unadjusted and adjusted logistic regression analysis of sociodemographic and clinical characteristics of death cases in severe patients with COVID-19/diabetes *mellitus* in Brazil from 2020 to 2022

Variables				DEATH		
	n	%	Unadjusted Odds Ratio (95%CI)	*p* value^*^	Adjusted Odds Ratio (95%CI)	*p* value^*^
Sex						
Female		41.9	1.00	-	-	-
Male	101,040	44.1	1.09 (1.08 - 1.11)	<0.001	1.06 (1.02-1.10)	0.002
Age						
Children (0 to 11 years)	43	12.7	1.00		-	-
Adolescents (12 to 18 years)	47	11.4	0.88 (0.57 - 1.37)	0.592	0.94 (0.33-2.67)	0.916
Adults (19 to 59 years)	44,039	31.1	3.10 (2.25 - 4.27)	<0.001	5.81 (2.51-13.)	<0.001
Older adults (over 60 years)	149,479	48.6	6.51 (4.73 - 8.97)	<0.001	12.37 (5.35-28.63)	<0.001
Pregnant woman						
1st trimester	11	13.3	1.00	-	-	-
2nd trimester	51	17.3	1.37 (0.68 - 2.77)	0.376	1.12 (0.46-2.70)	0.799
3rd trimester	77	10.3	0.75 (0.38 - 1.47)	0.405	0.47 (0.20-1.11)	0.087
Gestational age unknown	14	18.4	1.47 (0.62 - 3.49)	0.373	0.65 (0.17-2.51)	0.536
No	67,703	41.6	4.66 (2.47 - 8.79)	<0.001	1.64 (0.75-3.58)	0.214
Not applicable	118,681	44.4	5.23 (2.77 - 9.86)	<0.001	1.88 (0.86-4.11)	0.112
Race/color						
White	85,596	43.1	1.00	-	-	-
Black	11,026	48.3	1.23 (1.19 - 1.26)	<0.001	1.25 (1.20 - 1.31)	<0.001
Yellow	1,932	41.5	0.93 (0.88 - 0.99)	0.029	0.96 (0.87 - 1.07)	0.555
Brown	66,307	45.1	1.08 (1.06 - 1.09)	<0.001	1.23 (1.21 - 1.26)	<0.001
Indigenous	346	47.7	1.20 (1.03 - 1.39)	0.014	1.44 (1.14 - 1.81)	0.002
Education						
No education/illiterate	7,673	56.3	2.04 (1.95 - 2.14)	<0.001	1.56 (1.49 - 1.64)	<0.001
Elementary school 1st cycle (1st to 5th grade)	27,764	49.3	1.54 (1.49 - 1.59)	<0.001	1.29 (1.25 - 1.34)	<0.001
Elementary school 2nd cycle (6th to 9th grade)	15,162	45.6	1.32 (1.28 - 1.37)	<0.001	1.24 (1.19 - 1.29)	<0.001
High school (1st to 3rd year)	17,855	40.9	1.09 (1.06 - 1.13)	<0.001	1.12 (1.08 - 1.16)	<0.001
Higher education	7,263	38.7	1.00	-	-	-
Area						
Urban	163,143	42.8	1.00	-	-	-
Rural	8,589	46.0	1.14 (1.10 - 1.17)	<0.001	0.94 (0.89 - 0.98)	0.013
Periurban	716	48.2	1.24 (1.12 - 1.37)	<0.001	1.17 (0.99 - 1.39)	0.055
Flu-like syndrome outbreak						
Yes	17,540	44.2	1.11 (1.08 - 1.14)	<0.001	1.03 (1.00 - 1.06)	0.041
No	37,047	41.5	1.00	-	-	-
Nosocomial case						
Yes	5,255	53.6	1.61 (1.54 - 1.67)	<0.001	2.41 (2.18 - 2.67)	<0.001
No	141,124	41.7	1.00		-	-
Works with poultry or pigs						
Yes	1,575	40.2	1.07 (1.01 - 1.15)	0.020	-	-
No	127,048	42.0	1.00	-	1.08 (0.94 - 1.23)	0.237
Postpartum woman						
Yes	144	27.8	1.00	-	-	-
No	115,104	42.4	1.90 (1.57 - 2.31)	<0.001	1.77 (1.41 - 2.22)	<0.001
Chronic cardiovascular disease						
Yes	116,695	45.7	1.34 (1.32 - 1.36)	<0.001	1.25 (1.23 - 1.28)	<0.001
No	44,203	38.4	1.00	-	-	-
Chronic hematologic disease						
Yes	1,737	48.9	1.31 (1.23 - 1.40)	<0.001	0.98 (0.90 - 1.07)	0.790
No	113,346	42.1	1.00	-	-	-
Chronic liver disease						
Yes	2,804	52.8	1.54 (1.46 - 1.63)	<0.001	-	-
No	112,216	42.0	1.00	-	1.25 (1.16 - 1.34)	<0.001
Asthma						
Yes	4,437	39.1	1.00	-	1.24 (1.18 - 1.30)	<0.001
No	110,967	42.2	1.14 (1.09 - 1.18)	<0.001	-	-
Neurological disease						
Yes	10,768	55.6	1.76 (1.71 - 1.81)	<0.001	-	
No	107,090	41.5	1.00	-	1.60 (1.54 - 1.66)	<0.001
Chronic lung disease						
Yes	10,171	54.1	1.65 (1.60 - 1.70)	<0.001	1.46 (1.41 - 1.52)	<0.001
No	107,690	41.6	1.00	-	-	
Immunodeficiency or immunodepression						
Yes	5,276	51.1	1.44 (1.39 - 1.50)	<0.001	1.25 (1.19 - 1.32)	<0.001
No	110,332	41.9	1.00	-	-	-
Chronic kidney disease						
Yes	17,103	57.2	1.92 (1.87 - 1.96)	<0.001	1.73 (1.68 - 1.79)	<0.001
No	102,989	41.1	1.00	-	-	-
Obesity						
Yes	23,946	46.7	1.23 (1.21 - 1.25)	<0.001	1.04 (0.82 - 1.33)	0.699
No	95,880	41.5	1.00	-	-	-
Flu vaccine						
Yes	21,527	37.4	1.00	-	-	-
No	52,265	41.1	1.16 (1.14 - 1.19)	<0.001	0.97 (0.81 - 1.16)	0.810
Antiviral for flu						
Yes	15,101	42.3	1.04 (1.01 - 1.06)	0.001	0.77 (0.55 - 1.06)	0.117
No	118,764	41.4	1.00	-	-	-
Hospital/admission						
Yes	185,222	42.3	1.00	-	-	-
No	4,838	77.8	4.78 (4.50 - 5.08)	<0.001	4.78 (4.50 - 5.08)	<0.001
ICU						
Yes	114,685	63.8	5.14 (5.07 - 5.21)	<0.001	2.51 (2.10 - 3.02)	<0.001
No	57,259	25.5	1.00	-	-	-
Ventilatory support						
Yes	86,623	83.2	11.28 (11.07 - 11.49)	<0.001	7.79 (6.29 - 9.65)	<0.001
No	70,257	30.6	1.00	-	-	-
Closing criteria						
Laboratory	176,280	43.2	1.00	-	-	-
Link/epidemiological	2,087	45.4	1.09 (1.03 - 1.16)	0.003	2.80 (0.77 - 10.17)	0.116
Clinical	4,016	44.0	1.03 (0.99 - 1.07)	0.118	1.36 (0.53 - 3.49)	0.519

* *To validate the model, the Hosmer-Lemeshow test (p=0.08) and Nagelkerke (0.36) were performed, indicating the quality and fit of the model; ICU - Intensive Care Unit.*

In adjusted analysis, statistically significant associations were observed for the risk of death in people with COVID-19/DM for the following variables: male sex (OR=1.06; 95%CI 1.02-1.10); age group 19 to 59 years (OR=5.81; 95%CI 2.51-13); age over 60 years (OR=12.37; 95%CI 5.35-28.63); race/color black (OR=1.25; 95%CI 1.20-1.31); brown (OR=1.23; 95%CI 1.21-1.26); indigenous (OR=1.44; 95%CI 1.14 - 1.81); people with no education/illiterate (OR=1.56; 95%CI 1.49 - 1.64); elementary school 1^st^ cycle (1^st^ to 5^th^ grade) (OR= 1.29; 95%CI 1.25 - 1.34); elementary school 2^nd^ cycle (6^th^ to 9^th^ grade) (OR=1.24; 95%CI 1.19 - 1.29); and high school (OR=1.12; 95%CI 1.08 - 1.16). A statistically significant association was also identified with: deaths from nosocomial cases (OR=2.41; 95%CI 2.18-2.67); postpartum women (OR=1.77; 95%CI 1.41-2.22); chronic cardiovascular disease (OR=1.25; 95%CI 1.23-1.28); chronic liver disease (OR=1.25; 95%CI 1.16-1.34); asthma (OR=1.24; 95%CI 1.18-1.30); neurological disease (OR=1.60; 95%CI 1.54-1.66); chronic lung disease (OR=1.46; 95%CI 1.41-1.52); immunodeficiency or immunosuppression (OR=1.25; 95%CI 1.19-1.32); chronic kidney disease (OR=1.73; 95%CI 1.68-1.79); hospital/hospitalization (OR=4.78; 95%CI 4.50-5.08); ICU admission (OR=2.51; 95%CI 2.10-3.02); and ventilatory support (OR=7.79; 95%CI 6.29-9.65). The rural area variable (OR=0.94; 95%CI 0.89-0.98) was a protective factor.

Table 2 presents the results of unadjusted and adjusted logistic regression analysis of signs and symptoms, and tests performed. In adjusted analysis, the statistically significant variables associated with a higher risk of death in people with COVID-19/DM were dyspnea (OR=1.38; 95%CI 1.21-1.57), respiratory distress (OR=1.87; 95%CI 1.67-2.10), and SpO_2_<95% (OR=1.62; 95%CI 1.38-1.78). Among the radiological findings, interstitial infiltrate (OR=1.65; 95%CI 1.16-2.36), consolidation (OR=2.85; 95%CI1.80-4.54), mixed (OR=2.26; 95%CI1.42-3.61), other findings (OR=1.80; 95%CI1.23-2.63) and not performing the test (OR=1.52; 95%CI1.08-2.14). In addition, the indeterminate tomography aspect for COVID-19 (OR=1.40; 95%CI 1.01-1.93) and not performing the tomography (OR=0.53; 95%CI 0.32-0.89) also stood out as well as the immunofluorescence results (OR=1.66; 95%CI1.25-2.21). Variables such as cough (OR=0.73; 95%CI 0.65 - 0.2), sore throat (OR= 0.80; 95%CI 0.70 - 0.91) and antigen test not performed (OR=0.53; 95%CI 0.32 - 0.89) were shown to be protective factors.

**Table 2:** Unadjusted and adjusted logistic regression analysis of signs and symptoms, and tests performed on cases of death in severe patients with COVID-19/diabetes *mellitus* in Brazil from 2020 to 2022

Variables				DEATH		
	n	%	Unadjusted Odds Ratio (95%CI)	*p* value^*^	Adjusted Odds Ratio (95%CI)	*p* value^*^
Fever						
Yes	100,033	42.1	1.00	-	-	-
No	58,900	42.6	1.02 (1.00 - 1.03)	0.003	0.96 (0.87 - 1.07)	0.49
Cough						
Yes	125,522	40.8	0.80 (0.79 - 0.81)	<0.001	0.73 (0.65 - 0.82)	<0.001
No	40,209	46.2	1.00	-	-	-
Sore throat						
Yes	27,310	39.3	0.88 (0.86 - 0.89)	<0.001	0.80 (0.70 - 0.91)	<0.001
No	105,582	42.4	1.00	-	-	-
Dyspnea						
Yes	151,734	45.9	1.81 (1.78 - 1.84)	<0.001	1.38 (1.21 - 1.57)	<0.001
No	23,020	31.9	1.00	-	-	-
Respiratory distress						
Yes	125,182	47.7	1.82 (1.79 - 1.89)	<0.001	1.87 (1.67 - 2.10)	<0.001
No	36,103	33.4	1.00	-	-	-
SpO2<95%	2					
Yes	142,724	46.5	1.86 (1.83 - 1.89)	<0.001	1.62 (1.38 - 1.78)	<0.001
No	25,998	31.8	1.00	-	-	-
Diarrhea						
Yes	21,934	38.5	0.85 (0.83 - 0.86)	<0.001	0.98 (0.85 - 1.14)	0.83
No	110,320	42.5	1.00	-	-	-
Vomiting						
Yes	14,451	39.0	0.88 (0.86 - 0.90)	<0.001	0.93 (0.78 - 1.10)	0.38
No	115,560	42.2	1.00	-	-	-
Abdominal pain						
Yes	8,660	38.8	1.00	-	-	-
No	99,738	41.4	1.11 (1.08 - 1.14)	<0.001	1.02 (0.84 - 1.24)	0.82
Fatigue						
Yes	41,830	41.1	1.00	-	-	-
No	73,827	41.4	1.01 (0.99 - 1.02)	0.137	1.15 (1.03 - 1.29)	0.01
Loss of smell						
Yes	11,305	33.6	1.00	-	-	-
No	96,999	42.0	1.43 (1.39 - 1.46)	<0.001	1.13 (0.89 - 1.44)	0.31
Loss of taste						
Yes	11,491	33.3	1.00	-	-	-
No	96,779	42.0	1.45 (1.41 - 1.48)	<0.001	1.27 (1.00 - 1.61)	0.054
Chest X-ray						
Normal	2,575	34.3	1.00	-	-	-
Interstitial infiltrate	27,078	44.7	1.55 (1.47 - 1.63)	<0.001	1.65 (1.16 - 2.36)	0.005
Consolidation	4,290	53.2	2.17 (2.04 - 2.32)	<0.001	2.85 (1.80 - 4.54)	<0.001
Mixed	4,633	50.0	1.91 (1.79 - 2.03)	<0.001	2.26 (1.42 -3.61)	0.001
Other	12,892	45.0	1.56 (1.48 - 1.64)	<0.001	1.80 (1.23 - 2.63)	0.002
Not performed	49,870	39.2	1.23 (1.17 - 1.29)	<0.001	1.52 (1.08 - 2.14)	0.02
Type of clinical sample collected for testing						
Naso-oropharyngeal secretion	156,948	42.9	1.00	-	-	-
Brochoalveolar lavage	1,025	65.0	2.47 (2.23 - 2.75)	<0.001	1.81 (0.07 - 42.63)	0.713
Post-mortem tissue	77	80.2	5.40 (3.26 - 8.92)	<0.001	0.67 (0.54 - 0.84)	0.001
PCR						
Detectable	133,063	44.1	1.17 (1.14 - 1.19)	<0.001	1.23 (0.98 - 1.56)	0.08
Not detectable	8,642	33.0	0.73 (0.70 - 0.75)	<0.001	0.77 (0.57 - 1.03)	0.08
Inconclusive	330	39.2	0.95 (0.83 - 1.10)	0.546	1.17 (0.33 - 4.11)	0.80
Not performed	18,478	44.2	1.17 (1.14 - 1.20)	<0.001	1.05 (0.85 - 1.31)	0.63
Awaiting results	17,685	40.3	1.00	-	-	-
Tomography appearance						
Typical COVID-19	65,353	38.6	1.00	-	-	-
Undetermined COVID-19	3,467	42.6	1.18 (1.12 - 1.23)	<0.001	1.40 (1.01 - 1.93)	0.046
Atypical COVID-19	1,749	40.4	1.07 (1.01 - 1.14)	0.018	0.92 (0.65 - 1.31)	0.64
Negative for pneumonia	255	26.3	0.56 (0.49 - 0.65)	<0.001	0.40 (0.13 -1.27)	0.12
Not performed	30,244	47.5	1.44 (1.41 - 1.46)	<0.001	1.20 (1.08 - 1.33)	<0.001
Type of antigenic test						
Immunofluorescence	30,925	46.9	1.14 (1.12 - 1.17)	<0.001	1.66 (1.25 - 2.21)	<0.001
Rapid antigen test	33,372	43.5	1.00	-	-	-
Antigenic test result						
Positive	36,406	44.0	1.10 (1.09 - 1.12)	<0.001	1.14 (0.70 - 1.84)	0.60
Negative	2,596	37.8	0.85 (0.81 - 0.90)	<0.001	0.63 (0.38 - 1.05)	0.07
Inconclusive	17	43.6	1.09 (0.58 - 2.05)	0.785	1.73 (0.26 - 11.43)	0.57
Not performed	52,442	43.6	1.09 (1.07 - 1.10)	<0.001	0.53 (0.32 - 0.89)	0.02
Waiting for results	74,332	41.4	1.00	-	-	-
Serological test (IgM)						
Positive	13,146	43.5	1.00	-	-	-
Negative	4,347	47.1	1.15 (1.10 - 1.21)	<0.001	1.20 (0.97 - 1.49)	0.09
Inconclusive	48	53.3	1.48 (0.97 - 2.24)	0.063	1.54 (0.62 - 3.79)	0.35
Not performed	11,713	45.5	1.08 (1.04 - 1.12)	<0.001	0.96 (0.83 - 1.10)	0.51
Waiting for results	26	44.8	1.05 (0.62 - 1.76)	0.842	1.28 (0.89 - 2.31)	0.08

* *To validate the model, the Hosmer-Lemeshow test (p=0.11) and Nagelkerke (0.29) were performed, indicating the quality and fit of the model; SpO2 - peripheral oxygen saturation.*

## DISCUSSION

DM increases the risk of fatal complications in COVID-19^(19)^. In this regard, research conducted in Brazil showed that three out of every 20 deaths from COVID-19 were observed in people with DM, with a prevalence 1.15 times higher among hospitalized cases with DM compared to those without DM^(20)^. When analyzing the interconnection of comorbidity with sociodemographic factors, it was found that the risk of mortality was accentuated in males. This may be related to specific biological factors, considering that the almost three times greater expression of Angiotensin Converting Enzyme 2 in men, compared to women, contributes to the worsening of the disease, as the COVID-19 virus uses this enzyme as a receptor to enter cells^(21,22)^. In addition to biological aspects, cultural barriers such as the construction of masculinity play a significant role in men’s resistance to seeking healthcare services and lower adherence to preventive practices, which makes this population more vulnerable to illness^(23,24)^. These factors exemplify how structural and intermediate determinants, such as sex, cultural patterns, and health practices, influence the risk and outcome of COVID-19 in people with DM.

In terms of age group, the increased risk of mortality was present in adults and older adults. In this regard, older adults, especially those with pre-existing health conditions, have a higher risk of developing severe forms and death related to COVID-19 than younger people^(25)^. A study carried out in Brazil with the general population found that older adults aged 60 to 79 years had a 2.87 times greater risk of mortality from COVID-19, while older adults aged 80 years or older had a 7.06 times greater risk compared to individuals under 59 years old and, when associated with comorbidities, the probability of death increased by 9.44 times in relation to individuals without pre-existing health conditions^(26)^.

This condition can be explained by the progressive deterioration of the immune system with aging, which increases vulnerability to infectious diseases, making prognosis unfavorable if combined with chronic conditions^(27)^. It is worth noting that the increased risk of death observed in the adult population of this study may be associated with the fact that these individuals had DM, which may have increased the chances of mortality in this age group. Thus, age, as a structural determinant, interacts with intermediate determinants, such as the presence of comorbidities, potentiating negative outcomes.

In this study, brown, black and indigenous races were associated with an increased risk of death in severe patients with COVID-19/DM. Similarly, a study conducted in the United Kingdom, which assessed the association between race/color and survival in patients hospitalized with COVID-19, identified higher mortality rates in individuals of non-white ethnicity compared to those of white ethnicity^(28)^. A Brazilian study found that non-white skin color increased the risk of death from COVID-19 by 13%^(26)^.

The predominance of notifications among brown individuals in the sample results from Brazil’s racial diversity, which is characterized by a significant portion of people who self-declare as brown^(29)^. With regard to black race, they face a double vulnerability, because in addition to the prevalence of diagnoses of chronic diseases, such as DM, this population also faces challenges in accessing basic services, such as health, due to social barriers, such as inequality^(30)^. This vulnerability to COVID-19 is equally evident among indigenous peoples, as they face challenges such as resource scarcity, poverty and poor health. Thus, race and color constitute an important structural determinant, associated with precarious living and health conditions, which amplifies exposure to intermediate determinants and barriers to access to healthcare.

It is important to highlight that this inequality can be understood in light of racism, which acts as a structural determinant in influencing the health-disease process^(14)^. Non-white populations are disproportionately represented among groups with lower income, lower education levels, and poor access to healthcare services, which affects the virus transmission and increases complications related to COVID-19^(31)^.

In this study, different levels of education were associated with the occurrence of death; however, the greatest chance was indicated for those with no education/illiteracy. In this regard, a Brazilian study also identified low education as a sociodemographic risk factor for the outcome of death in cases of SARS due to COVID-19^(32)^. This indicates that low education levels may represent a barrier to access to information, resulting in insufficient knowledge about the disease, which may lead to difficulties in control and self-care, especially in people with DM, where these difficulties may directly affect glycemic control and cause changes in behavior related to self-care^(33)^. In this context, education is a fundamental structural determinant, as it directly influences access to information, income and healthcare services, in addition to impacting the ability to understand and adopt self-care practices that are important in the management of chronic diseases such as DM. These factors, in turn, affect intermediate determinants and contribute to the widening of health inequalities.

The high death rate associated with nosocomial cases supports research conducted in Sweden, in which SARS-CoV-2 infection acquired in hospitals during the early stages of the pandemic resulted in a significant increase in the risk of mortality^(34)^. In the context of DM, susceptibility to hospital infections can be explained by the impairment of the immune system. This condition compromises the body’s ability to resist any infection, making these people more vulnerable^(8)^. This scenario highlights the complex interaction between intermediate determinants, such as the presence of comorbidities, and factors related to the type of care received, such as hospitalization and ICU support, which directly influence the severity of clinical condition and health outcomes, such as the occurrence of death.

In addition, chronic cardiovascular diseases, asthma, chronic kidney disease and chronic lung disease have been identified as risk factors for death in critically ill patients with COVID-19/DM. In this regard, NCDs are emerging as a global health problem that contributes to a significant increase in the number of premature deaths, standing out as a priority in the 2030 Agenda proposed by the United Nations (UN)^(16)^. In Brazil, deaths from NCDs increased by 49.6%, totaling 337,098 deaths in 2019^(35)^. Furthermore, the intersection between NCDs and COVID-19 increases the vulnerability of these patients, because the prognosis of people with pre-existing comorbidities is unfavorable when compared to that of physically healthy populations^(36)^. These conditions constitute intermediate determinants, as they are directly related to the health status of individuals. When combined with other vulnerabilities, such as difficulties in accessing healthcare services or poor quality of care received, these non-communicable conditions increase the risks, by negatively influencing the clinical course and increasing the probability of death in severe patients with COVID-19/DM.

Another important aspect to be mentioned is that, in this research, people with DM who are immunodeficient or immunosuppressed had a high risk of mortality from SARS due to COVID-19. The literature reinforces this association, indicating that patients with two or more comorbidities are more likely to die from COVID-19 than those with a single comorbidity or none. Moreover, COVID-19 infection itself is known to dysregulate the immune system, contributing to a worse prognosis^(37)^.

This study also showed that people with DM who were not previously hospitalized/admitted had a higher chance of death. Despite this result, the literature highlights that people with DM are more likely to be hospitalized for SARS due to COVID-19, as they have an increased risk of developing more severe forms of the disease^(38)^. These findings may be attributed to delays and hesitation in seeking medical care. In this regard, the spread of false and inaccurate news about the disease, clinical severity, and treatment options not supported by clinical data, especially early in the pandemic, may have led to confusion and risky behaviors^(39)^. These findings reveal important difficulties related to timely access and quality of healthcare, which act as mediating elements between structural determinants (such as education, income and place of residence) and intermediate determinants (such as the presence of comorbidities and clinical symptoms). The way these factors are managed in the healthcare system can directly influence clinical outcomes, such as disease severity and risk of death.

A relationship was also found between the need for ventilatory support and ICU admission and deaths in critically ill patients with DM and COVID-19. This is because ICU admission and invasive or non-invasive ventilatory support are indicative of a critical condition of SARS^(40)^. In addition, metabolic changes, such as decreased immune response and increased inflammation in people with DM, increase the risk of serious complications and mortality from COVID-19^(41)^. These factors represent intermediate determinants of health, such as comorbidities and the severity of the clinical condition, which, when interacting with elements of healthcare, such as the time of initiation of treatment, the quality of care provided and the availability of hospital resources, can intensify patients’ vulnerability and negatively impact clinical outcomes.

In relation to the pathophysiological changes of DM, this condition not only alters lung function, but also increases the permeability of blood vessels and affects the epithelial cells of the alveoli. This fact may justify the greater occurrence of respiratory symptoms in fatal cases in people with DM affected by SARS due to COVID-19, such as dyspnea, respiratory distress and SpO_2_<95%^(42)^. These respiratory symptoms represent intermediate clinical determinants, as they reflect the severity of patients’ health condition at the time of care. Their presence is directly related to a more severe clinical condition, indicating greater lung involvement, which increases the risk of complications and death.

In general, DM patients who died from severe COVID-19 underwent serological tests, including RT-PCR and immunofluorescence testing to identify the presence of the virus. According to COVID-19 diagnostic protocols, the use of molecular tests, such as Quantitative Reverse Transcription PCR, is recommended as the reference method for diagnosing the disease in Brazil, due to its high accuracy and reliability^(4)^. In this investigation, abnormal radiological findings on chest X-ray, indeterminate appearance for COVID-19 on tomography and not performing this test showed a statistically significant association with the outcome of death. This reinforces the importance of imaging tests in COVID-19 diagnosis, since not performing this type of test compromises, especially in more severe cases, the analysis of the extent of the disease, alternative diagnoses and identification of complications^(43)^.

The availability and effective performance of diagnostic tests, such as chest X-rays and CT scans, are fundamental elements of healthcare, as they directly influence the accuracy of diagnosis, the definition of the severity of the clinical condition and therapeutic decision-making. When these tests are not performed, especially in severe cases such as those resulting from SARS-CoV-2 infection, there is a limitation in medical assessment, which can delay appropriate interventions and increase the risk of unfavorable outcomes, such as death.

It is important to note that, in this study, the variables that in the final regression model were presented as protective factors for death were living in a rural area, having a cough, sore throat and not having undergone the antigen test. It is believed that the association between rural areas and a lower risk of death is related to the lower population density; thus, in rural areas, there would be less transmission of the virus, unlike what occurs in metropolitan regions^(44)^. However, the area of residence, as a structural determinant, can also influence access to specialized healthcare services, representing both protective and limiting factors, depending on the context being analyzed. Despite being serious patients, symptoms such as cough and sore throat can act as a protective factor, suggesting that the infection is still restricted to the upper airways, with a less systemic inflammatory response, which favors early diagnosis and treatment^(45)^. As for the non-performance of the antigenic test, this variable may reflect access to other more sensitive diagnostic methods, favoring the appropriate management of the case and better prognoses.

Although statistically significant associations were identified between the deaths of severe patients with COVID-19/DM and variables such as not having recently given birth, not having chronic liver disease and neurological disease, it is worth noting that the data analyzed did not provide specific information on each associated characteristic in isolation. Therefore, it is possible to consider that these patients could have presented other risk factors not covered in the study, which could contribute to the death outcome.

### Study limitations

As a limitation, it is worth noting that, since this is a study with secondary data, there is a possibility of underreporting of data and incorrect completion by the professionals executing the notification. Another important factor is the “ignored” option available in the variables filled in on the form, which makes it impossible to interpret the data in detail.

### Contributions to nursing, health or public policy

Epidemiological studies are important because they provide the scientific basis necessary for adapting nursing practices, directly influencing the formulation of public health policies aimed at controlling the disease and monitoring its evolution, especially in the face of emerging challenges. Furthermore, they directly contribute to achieving the goals set by the 2030 Agenda by aligning these practices with the UN SDG “Health and Well-being” to promote a healthier future. By understanding these epidemiological patterns associated with DM, nursing professionals can improve prevention, control and treatment skills targeted at this population, aiming to reduce the prevalence of DM and improve patients’ quality of life, together with the planning and implementation of actions for future health crises.

## CONCLUSIONS

Based on the study, it was possible to verify that sociodemographic, clinical and care factors were associated with death in severe patients with COVID-19/DM such as: male sex; age range from 19 to 59 years; older adults; non-white race/color; low education level; nosocomial cases; ICU admission; ventilatory support; chronic cardiovascular diseases; asthma; chronic lung diseases; immunosuppression and chronic kidney diseases; respiratory distress; dyspnea; saturation <95%; RT-PCR tests; immunofluorescence; tomography aspect indeterminate for COVID-19; atypical for COVID-19; negative for pneumonia; and not performed. The rural area variable was shown to be a protective factor. In this sense, the complexity of this comorbidity is highlighted, emphasizing the importance of interventions targeted at more vulnerable groups and the need for effective prevention, monitoring, and treatment strategies to reduce the fatal impact of this interaction.

These results reflect the complex interrelationship between structural and intermediate determinants of health, as per the SDH model. In this context, interventions targeting more vulnerable groups, based on SDH, are essential to reduce inequalities in access to health and promote equity in treatment. Effective prevention, monitoring and treatment strategies must be designed taking into account these determinants, in order to mitigate the fatal impact of the interaction between DM and COVID-19, especially in populations in situations of greater social and health vulnerability.

## Data Availability

The research data are available in a repository: https://doi.org/10.48331/scielodata.0RJPTY.
