# Supplementary material for: Factors associated with death in severe patients with COVID-19/diabetes mellitus in Brazil
Source: Rev Bras Enferm. 2025 Dec 15;78(5):e20240361. doi: 10.1590/0034-7167-2024-0361 (PMC12707847; doi:10.1590/0034-7167-2024-0361)
Supplement: Translation

# Translation: Fatores associados ao óbito em pacientes graves com COVID-19/diabetes *mellitus* no Brasil

**DOI:** 10.1590/0034-7167-2024-0361pt

**Authors:** Isabella Rodrigues da Silva Batista Lima, Andressa Jhulier Faiola Oliveira, Lívia Maia Pascoal, Floriacy Stabnow Santos, Leonardo Hunaldo dos Santos, Marcelino Santos, Janaina Miranda Bezerra, Ana Cristina Pereira de Jesus Costa

**Affiliations:** I Universidade Federal do Maranhão. Imperatriz, Maranhão, Brasil

**Keywords:** Diabetes *Mellitus*, COVID-19, Síndrome Respiratória Aguda Grave, Fatores de Risco, Mortalidade.

## Abstract

**Objetivos:**

analisar os fatores associados ao óbito em pacientes graves com COVID-19/diabetes *mellitus* no Brasil.

**Métodos:**

estudo transversal, realizado com notificações de doenças respiratórias graves por COVID-19 registradas no banco de dados do Sistema Único de Saúde, no período entre 2020 e 2022, Brasil. Para analisar os fatores associados, utilizaram-se modelos de regressão logística univariada e multivariada.

**Resultados:**

pessoas do sexo masculino, faixa 19 a 59 anos, idosos, raça/cor não branca, baixa escolaridade, casos nosocomiais, internação em Unidade de Terapia Intensiva, suporte ventilatório, doenças cardiovasculares e renais crônicas, asma, pneumopatias crônicas, imunodepressão, manifestações clínicas, exames sorológicos, e imagem se associaram ao maior risco de óbito. As variáveis zona rural, tosse, dor de garganta e teste antigênico não realizado foram fatores de proteção.

**Conclusões:**

esses dados reforçam a necessidade de estratégias baseadas nos determinantes sociais da saúde para ampliar o acesso, manejo clínico e reduzir a mortalidade nesses pacientes.

## INTRODUÇÃO

A COVID-19, doença causada pela infecção por SARS-CoV-2, é uma enfermidade respiratória aguda grave com alto potencial de transmissibilidade. Além do acometimento respiratório, a COVID-19 pode ocasionar comprometimento multissistêmico e prolongado da doença em alguns indivíduos^(1,2)^. Segundo o relatório da Organização Pan-Americana de Saúde, durante a 30ª Conferência Sanitária Pan-Americana, que ocorreu em setembro de 2022, a região das Américas foi responsável pela notificação de 30% e 44% dos casos, e mortes por COVID-19 no mundo, respectivamente. Entre os países da América, os Estados Unidos da América e o Brasil se destacaram entre os dez países com maior número de casos acumulados em escala mundial^(3)^.

Os sintomas associados à infecção por COVID-19 podem variar de leves a graves, incluindo a síndrome respiratória aguda grave (SRAG), que é caracterizada pela saturação periférica de oxigênio (SpO_2_) <95%, hipotensão, desconforto respiratório e piora nas condições clínicas de doenças pré-existentes^(4)^. Desde os primeiros relatos, muitos casos graves e fatais de COVID-19 ocorreram em pacientes idosos ou pessoas com comorbidades, especialmente aqueles com diabetes *mellitus* (DM), hipertensão arterial sistêmica, doenças cerebrovasculares, doenças renais e pulmonares crônicas^(5)^. Destaca-se, ainda, que pessoas com DM apresentam risco 2,3 vezes maior de desenvolver formas mais graves da doença, além de apresentarem um risco 2,5 vezes maior de mortalidade relacionada à COVID-19^(6)^.

O DM emerge como um desafio crescente à saúde pública, conforme apontam as projeções da Federação Internacional de Diabetes. Cerca de 537 milhões de adultos com idades entre 20 e 79 anos em todo o mundo possuem essa condição, representando 10,5% da população global. Estimativas sugerem um aumento significativo de 46% de pessoas vivendo com o DM até o ano de 2045. Além disso, esse aumento, durante o mesmo período, será mais acentuado em nações caracterizadas por baixa e média renda, refletindo o crescimento populacional nessas regiões^(7)^.

A relação entre o DM e outras doenças infecciosas pode ser atribuída à dificuldade das pessoas com DM apresentarem resposta imunológica eficaz contra infecções, o que aumenta sua vulnerabilidade e predisposição para o desenvolvimento de doença ativa. Devido a essa condição, pessoas com diabetes são classificadas como grupo de risco elevado para doenças infecciosas, incluindo a infecção pelo SARS-CoV-2^(8)^, contudo ainda existem lacunas sobre essa interrelação. A literatura aponta que existe uma ligação bidirecional entre a fisiopatologia do DM e o vírus da COVID-19, de modo que o DM contribui para o desenvolvimento de complicações quando o paciente é infectado pelo coronavírus^(9)^. Associado a essa complexa relação, é possível considerar que o DM contribui para resultados clínicos desfavoráveis, elevando o risco para desfecho de óbito^(10)^. Além disso, a hiperglicemia na admissão hospitalar foi apontada como indicador de mortalidade por todas as causas em pacientes hospitalizados com COVID-19 que não estavam em estado crítico, independentemente da existência prévia de histórico de diabetes^(11)^.

No que se refere às complicações, estudo conduzido em um hospital de referência para casos graves de COVID-19 na cidade de Jacarta, Indonésia, revelou que o DM aumentou o risco de tratamento intensivo em 2,57 vezes, em comparação com aqueles que não tinham DM^(10)^. Outra pesquisa evidenciou que, no grupo de pessoas com DM, foram observadas complicações com maior frequência do que no grupo sem DM, e as mais prevalentes foram desconforto respiratório agudo, lesão cardíaca aguda e lesão renal aguda^(12)^.

Além dos fatores clínicos, características socioeconômicas como baixa escolaridade, raça/cor não branca e acesso limitado a serviços de saúde estão associadas ao aumento do risco de mortalidade nesse grupo populacional^(13)^. A inter-relação entre fatores sociais e biológicos evidencia a importância de se abordar a vulnerabilidade em saúde a partir de uma perspectiva multidimensional, conforme delineado pelos modelos dos determinantes sociais da saúde (DSS) da Organização Mundial da Saúde. Esses modelos evidenciam como as desigualdades estruturais moldam os desfechos clínicos e influenciam os determinantes de saúde de maneira complexa e interdependente^(14)^.

Considerando a potencial ocorrência de complicações incapacitantes da própria doença, além de infecções futuras que podem surgir e se tornar potencialmente fatais, o DM integra, como componente essencial, o Plano de Ações Estratégicas para o Enfrentamento das Doenças Crônicas e Agravos não Transmissíveis (DCNT) no Brasil, 2021-2030^(15)^. Destacado como uma prioridade para a promoção da saúde, o DM também está alinhado aos compromissos globais preconizados pelos Objetivos de Desenvolvimento Sustentável (ODS) da Agenda 2030, em particular ao ODS 3 (“Saúde e Bem-estar”). A meta 3.4 visa reduzir até 2030 um terço da mortalidade prematura por doenças crônicas não transmissíveis, reforçando a necessidade de abordar as complexidades das condições crônicas como o DM^(16)^.

Embora as evidências mostrem que as pessoas com DM correm o risco de contrair COVID-19, é importante conhecer os fatores associados ao óbito. Essa compreensão permite elaborar estratégias que previnam desfechos graves, facilitando a execução de ações direcionadas para prevenção e assistência adequada desses indivíduos. Devido à representatividade significativa desse grupo na população suscetível a complicações graves da doença, a interseção entre COVID-19 e o DM não só ressalta os desafios da saúde pública, mas também lança luz sobre questões fundamentais relacionadas ao bem-estar global.

## OBJETIVOS

Analisar os fatores associados aos óbitos em pacientes graves com COVID-19/DM no Brasil.

## MÉTODOS

### Aspectos éticos

O consentimento do paciente foi dispensado, uma vez que a pesquisa se baseia unicamente em dados secundários disponíveis no *OpenDATASUS*. Essa plataforma governamental oferece acesso abrangente a dados de saúde no Brasil de forma pública e anonimizada, sem apresentar riscos para os participantes.

### Desenho, período e local do estudo

Trata-se de estudo com delineamento transversal, conduzido a partir do instrumento *STrengthening the Reporting of OBservational studies in Epidemiology*, realizado com dados notificados de SRAG no período de 25 de fevereiro de 2020 a 30 de setembro de 2022, no Brasil, país que fica localizado na América do Sul, que abriga uma população estimada de 213.759.869 habitantes, distribuída em 27 Unidades Federativas e cinco macrorregiões (Sul, Sudeste, Centro-Oeste Norte e Nordeste)^(17)^. Vale destacar que o período de início corresponde à notificação do primeiro caso de COVID-19 registrado no país.

### População ou amostra; critérios de inclusão e exclusão

A população foi constituída por todos os registros de SRAG devido à COVID-19 associados ao DM, notificados no banco de dados *OpenDATASUS* no período do estudo. Foram incluídas as notificações em que a pessoa apresentava DM (independentemente da classificação tipo 1 ou 2), sendo excluídas as notificações em que os pacientes não apresentaram resultado positivo para COVID-19. Foram considerados casos positivos para COVID-19 aqueles com confirmação do vírus SARS-CoV-2 na ficha de notificação, por meio de exames como biologia molecular reação em cadeia da polimerase em tempo real (RT-PCR), achados indicativos em raio-X e tomografia de tórax, além de testes rápidos para SARS-CoV-2 positivos para anticorpos IgM e/ou IgG. Informações incompletas na ficha de notificação que não especificaram se o paciente tinha DM, o desfecho do caso e notificações de óbitos fora do período de estudo foram desconsiderados.

### Protocolo do estudo

Neste estudo, os DSS foram adotados como modelo teórico por sua abordagem multidimensional, sendo expressos dois níveis interligados de determinação: os estruturais, ligados a condições sociodemográficas, e os intermediários, relacionados a aspectos clínico-biológicos, incorporando ainda o acesso e a qualidade dos serviços de saúde, o que permite analisar como tais fatores influenciam os óbitos em pacientes graves com SRAG por COVID-19 e DM no Brasil.

Na presente investigação, essas categorias foram operacionalizadas a partir das variáveis sociodemográficas e clínico-epidemiológicas extraídas do banco de dados analisado. Os determinantes estruturais (sexo, idade, raça/cor, escolaridade, zona de residência e situação gestacional) influenciam os determinantes intermediários (comorbidades e sinais e sintomas clínicos), os quais, por sua vez, interagem dinamicamente com os fatores relacionados ao acesso e à atenção em saúde (histórico epidemiológico e tipo de atendimento recebido). A efetividade do cuidado é mensurada por meio de exames diagnósticos e critérios clínicos aplicados, os quais, em conjunto, contribuem para o desfecho de óbito por SRAG-COVID-19. Para estruturar a análise dessas relações, esta investigação adaptou o modelo teórico dos DSS proposto por Solar e Irwin (2010)^(14)^.

A coleta de dados ocorreu em de outubro de 2022, a partir de informações do banco de dados *OpenDATASUS* (opendatasus.saude.gov.br/dataset/bd-srag-2021), vinculado ao Ministério da Saúde. Selecionaram-se variáveis clínicas e sociodemográficas com desfecho de óbito de SRAG por COVID-19, sendo elas: sexo (masculino e feminino); idade (crianças: 0 a 11 anos; adolescentes: 12 a 18 anos; adultos 19 a 59 anos; e idosos: acima de 60 anos); gestante (1° trimestre, 2° trimestre, 3° trimestre, idade gestacional ignorada, não e não se aplica); raça/cor (branca, preta, amarela, parda e indígena); escolaridade (sem escolaridade, fundamental 1° ciclo: 1ª a 5ª série; fundamental 2° ciclo: 6ª a 9ª série; médio: 1° ao 3° ano; e superior); zona (urbana, rural e periurbana); fatores de risco/comorbidades (DM e doença cardiovascular crônica, doença hematológica crônica, puérpera, síndrome de *Down*, doença neurológica crônica, pneumopatia crônica, imunodeficiência ou imunodepressão, doença renal crônica, doença hepática crônica, asma e obesidade); sinais e sintomas (febre, tosse, dor de garganta, dispneia, desconforto respiratório, saturação de oxigênio, diarreia, vômito, dor abdominal, fadiga, perda de olfato e perda de paladar), dados de atendimento (surto de síndrome gripal, caso nosocomial, trabalha com aves e suíno, vacina contra gripe, antiviral para gripe, hospital/internação, Unidade de Terapia Intensiva (UTI) e suporte ventilatório); raio-X de tórax (normal, infiltrado intersticial, consolidação, misto, outro e não realizado); tipo de amostra coletada para teste (secreção de naso-orofaringe, lavado bronco-alveolar e tecido *post-mortem*); PCR (detectável, não detectável, inconclusivo, não realizado e aguardando resultado); aspecto da tomografia (típico COVID-19, indeterminado COVID-19, atípico COVID-19, negativo para pneumonia e não realizado); tipos de teste antigênico (imunofluorescência e teste rápido antigênico); resultado de teste antigênico (positivo, negativo, inconclusivo, não realizado e aguardando resultado); teste sorológico IgM (positivo, negativo, inconclusivo, não realizado e aguardando resultado); e critério de encerramento (laboratorial, vínculo/epidemiológico e clínico).

### Análise dos resultados e estatística

Após identificar possíveis erros e inconsistências, conduziu-se uma análise descritiva, no qual foram apresentadas frequências absolutas e relativas para as variáveis sociodemográficas e clínico-epidemiológicas. Para avaliar possíveis fatores de risco do óbito por SRAG devido à COVID-19 entre variáveis sociodemográficas e clínico-epidemiológicas, empregaram-se modelos de regressão logística simples e múltiplos^(18)^, considerando a resposta binária (óbito: sim ou não). Na seleção dos principais fatores de risco, a análise logística univariada (não ajustada) foi inicialmente realizada, com critério de seleção p<0,20.

A multicolinearidade de variáveis independentes foi testada com base no *variance inflation factor*, calculado por regressão linear múltipla, cujo ponto de corte adotado foi o de *variance inflation factor* > 10, não sendo observada multicolinearidade em nenhum dos casos. Testou-se, ainda, a presença de autocorrelação por meio do teste de Durbin-Watson para ambos os modelos, indicando homogeneidade de variância dos resíduos. Em seguida, as variáveis selecionadas foram submetidas à regressão logística multivariada (ajustada), utilizando o método de seleção passo a passo *backward*. A escolha do melhor modelo foi realizada com base no menor valor de *Akaike Information Criterion*. Calcularam-se, ainda, as Razões de Chances (*Odds Ratio*), acompanhadas por Intervalos de Confiança de 95%. Todos os testes foram realizados por meio do programa IBM *Statistical Package for the Social Sciences*, versão 24.0, com significância de 5%.

## RESULTADOS

No período de 2020 a 2022, foram notificados 3.381.018 casos de SRAG no Brasil. Após a aplicação dos critérios de inclusão e exclusão, 484.139 casos apresentavam COVID-19/DM, dos quais 193.591 evoluíram para óbito. A análise de regressão logística univariada e multivariada das características sociodemográficas e clínicas dos óbitos em pacientes graves com COVID-19/DM no Brasil de 2020 a 2022 pode ser observada na Tabela 1.

**Tabela 1:** Análise de regressão logística não ajustada e ajustada das características sociodemográficas e clínicas dos casos de óbito em pacientes graves com COVID-19/diabetes *mellitus* no Brasil de 2020 a 2022

Variáveis				ÓBITO		
	n	%	*Odds Ratio* não ajustada (IC95%)	Valor de p^*^	*Odds Ratio* ajustada (IC95%)	Valor de p^*^
Sexo						
Feminino	92.551	41,9	1,00	-	-	-
Masculino	101.040	44,1	1,09 (1,08 - 1,11)	<0,001	1,06 (1,02-1,10)	0,002
Idade						
Crianças (0 a 11 anos)	43	12,7	1,00		-	-
Adolescentes (12 a 18 anos)	47	11,4	0,88 (0,57 - 1,37)	0,592	0,94 (0,33-2,67)	0,916
Adultos (19 a 59 anos)	44.039	31,1	3,10 (2,25 - 4,27)	<0,001	5,81 (2,51-13,)	<0,001
Idosos (acima de 60 anos)	149.479	48,6	6,51 (4,73 - 8,97)	<0,001	12,37 (5,35-28,63)	<0,001
Gestante						
1º trimestre	11	13,3	1,00	-	-	-
2º trimestre	51	17,3	1,37 (0,68 - 2,77)	0,376	1,12 (0,46-2,70)	0,799
3º trimestre	77	10,3	0,75 (0,38 - 1,47)	0,405	0,47 (0,20-1,11)	0,087
Idade gestacional ignorada	14	18,4	1,47 (0,62 - 3,49)	0,373	0,65 (0,17-2,51)	0,536
Não	67.703	41,6	4,66 (2,47 - 8,79)	<0,001	1,64 (0,75-3,58)	0,214
Não se aplica	118.681	44,4	5,23 (2,77 - 9,86)	<0,001	1,88 (0,86-4,11)	0,112
Raça/cor						
Branca	85.596	43,1	1,00	-	-	-
Preta	11.026	48,3	1,23 (1,19 - 1,26)	<0,001	1,25 (1,20 - 1,31)	<0,001
Amarela	1.932	41,5	0,93 (0,88 - 0,99)	0,029	0,96 (0,87 - 1,07)	0,555
Parda	66.307	45,1	1,08 (1,06 - 1,09)	<0,001	1,23 (1,21 - 1,26)	<0,001
Indígena	346	47,7	1,20 (1,03 - 1,39)	0,014	1,44 (1,14 - 1,81)	0,002
Escolaridade						
Sem escolaridade/analfabeto	7.673	56,3	2,04 (1,95 - 2,14)	<0,001	1,56 (1,49 - 1,64)	<0,001
Fundamental 1º ciclo (1ª a 5ª série)	27.764	49,3	1,54 (1,49 - 1,59)	<0,001	1,29 (1,25 - 1,34)	<0,001
Fundamental 2º ciclo (6ª a 9ª série)	15.162	45,6	1,32 (1,28 - 1,37)	<0,001	1,24 (1,19 - 1,29)	<0,001
Médio (1º ao 3º ano)	17.855	40,9	1,09 (1,06 - 1,13)	<0,001	1,12 (1,08 - 1,16)	<0,001
Superior	7.263	38,7	1,00	-	-	-
Zona						
Urbana	163.143	42,8	1,00	-	-	-
Rural	8.589	46,0	1,14 (1,10 - 1,17)	<0,001	0,94 (0,89 - 0,98)	0,013
Periurbana	716	48,2	1,24 (1,12 - 1,37)	<0,001	1,17 (0,99 - 1,39)	0,055
Surto de síndrome gripal						
Sim	17.540	44,2	1,11 (1,08 - 1,14)	<0,001	1,03 (1,00 - 1,06)	0,041
Não	37.047	41,5	1,00	-	-	-
Caso nosocomial						
Sim	5.255	53,6	1,61 (1,54 - 1,67)	<0,001	2,41 (2,18 - 2,67)	<0,001
Não	141.124	41,7	1,00		-	-
Trabalha com aves ou suínos						
Sim	1.575	40,2	1,07 (1,01 - 1,15)	0,020	-	-
Não	127.048	42,0	1,00	-	1,08 (0,94 - 1,23)	0,237
Puérpera						
Sim	144	27,8	1,00	-	-	-
Não	115.104	42,4	1,90 (1,57 - 2,31)	<0,001	1,77 (1,41 - 2,22)	<0,001
Doença cardiovascular crônica						
Sim	116.695	45,7	1,34 (1,32 - 1,36)	<0,001	1,25 (1,23 - 1,28)	<0,001
Não	44.203	38,4	1,00	-	-	-
Doença hematológica crônica						
Sim	1.737	48,9	1,31 (1,23 - 1,40)	<0,001	0,98 (0,90 - 1,07)	0,790
Não	113.346	42,1	1,00	-	-	-
Doença hepática crônica						
Sim	2.804	52,8	1,54 (1,46 - 1,63)	<0,001	-	-
Não	112.216	42,0	1,00	-	1,25 (1,16 - 1,34)	<0,001
Asma						
Sim	4.437	39,1	1,00	-	1,24 (1,18 - 1,30)	<0,001
Não	110.967	42,2	1,14 (1,09 - 1,18)	<0,001	-	-
Doença neurológica						
Sim	10.768	55,6	1,76 (1,71 - 1,81)	<0,001	-	
Não	107.090	41,5	1,00	-	1,60 (1,54 - 1,66)	<0,001
Pneumopatia crônica						
Sim	10.171	54,1	1,65 (1,60 - 1,70)	<0,001	1,46 (1,41 - 1,52)	<0,001
Não	107.690	41,6	1,00	-	-	
Imunodeficiência ou imunodepressão						
Sim	5.276	51,1	1,44 (1,39 - 1,50)	<0,001	1,25 (1,19 - 1,32)	<0,001
Não	110.332	41,9	1,00	-	-	-
Doença renal crônica						
Sim	17.103	57,2	1,92 (1,87 - 1,96)	<0,001	1,73 (1,68 - 1,79)	<0,001
Não	102.989	41,1	1,00	-	-	-
Obesidade						
Sim	23.946	46,7	1,23 (1,21 - 1,25)	<0,001	1,04 (0,82 - 1,33)	0,699
Não	95.880	41,5	1,00	-	-	-
Vacina contra gripe						
Sim	21.527	37,4	1,00	-	-	-
Não	52.265	41,1	1,16 (1,14 - 1,19)	<0,001	0,97 (0,81 - 1,16)	0,810
Antiviral para gripe						
Sim	15.101	42,3	1,04 (1,01 - 1,06)	0,001	0,77 (0,55 - 1,06)	0,117
Não	118.764	41,4	1,00	-	-	-
Hospital/internação						
Sim	185.222	42,3	1,00	-	-	-
Não	4.838	77,8	4,78 (4,50 - 5,08)	<0,001	4,78 (4,50 - 5,08)	<0,001
UTI						
Sim	114.685	63,8	5,14 (5,07 - 5,21)	<0,001	2,51 (2,10 - 3,02)	<0,001
Não	57.259	25,5	1,00	-	-	-
Suporte ventilatório						
Sim	86.623	83,2	11,28 (11,07 - 11,49)	<0,001	7,79 (6,29 - 9,65)	<0,001
Não	70.257	30,6	1,00	-	-	-
Critério de encerramento						
Laboratorial	176.280	43,2	1,00	-	-	-
Vínculo/epidemiológico	2.087	45,4	1,09 (1,03 - 1,16)	0,003	2,80 (0,77 - 10,17)	0,116
Clínico	4.016	44,0	1,03 (0,99 - 1,07)	0,118	1,36 (0,53 - 3,49)	0,519

* *Para validação do modelo, foi realizado o teste de Hosmer-Lemeshow (p=0,08), Nagelkerke (0,36), indicando qualidade e ajuste do modelo; UTI - Unidade de Terapia Intensiva.*

Na análise ajustada, observaram-se associações estatisticamente significantes para o risco de óbito em pessoas com COVID-19/DM para as seguintes variáveis: sexo masculino (OR=1,06; IC95%1,02-1,10); faixa etária 19 a 59 anos (OR=5,81 IC95% 2,51-13); idade acima de 60 anos (OR=12,37; IC95% 5,35-28,63); raça/cor preta (OR=1,25; IC95% 1,20 - 1,31); parda (OR=1,23; IC95% 1,21 - 1,26); indígena (OR=1,44; IC95% 1,14 - 1,81); pessoas sem escolaridade/analfabetos (OR=1,56; IC95% 1,49 - 1,64); fundamental 1º ciclo (1ª a 5ª série) (OR= 1,29; IC95% 1,25 - 1,34); fundamental 2º ciclo (6ª a 9ª série) (OR=1,24; IC95% 1,19 - 1,29); e médio (OR=1,12; IC95% 1,08 - 1,16). Identificou-se, ainda, associação estatisticamente significante com: óbitos provenientes de casos nosocomial (OR=2,41; IC95% 2,18 - 2,67); puérpera (OR=1,77; IC95% 1,41 - 2,22); doença cardiovascular crônica (OR= 1,25; IC95% 1,23 - 1,28); doença hepática crônica (OR=1,25; IC95% 1,16 - 1,34); asma (OR=1,24; IC95%1,18 - 1,30); doença neurológica (OR=1,60; IC95% 1,54 - 1,66); pneumopatia crônica (OR=1,46; IC95% 1,41 - 1,52); imunodeficiência ou imunodepressão (OR= 1,25; IC95% 1,19 - 1,32); doença renal crônica (OR=1,73; IC95% 1,68 - 1,79); hospital/internação (OR=4,78; IC95% 4,50 - 5,08); internação em UTI (OR= 2,51; IC95% 2,10 - 3,02); e suporte ventilatório (OR= 7,79; IC95% 6,29 - 9,65). A variável zona rural (OR= 0,94; IC95% 0,89 - 0,98) apresentou-se como fator de proteção.

A Tabela 2 apresenta os resultados da análise de regressão logística não ajustada e ajustada dos sinais e sintomas, e exames realizados. Na análise ajustada, as variáveis estatisticamente significativas associadas a maior risco de óbito em pessoas com COVID-19/DM foram dispneia (OR=1,38; IC95% 1,21 - 1,57), desconforto respiratório (OR=1,87; IC95% 1,67 - 2,10) e SpO_2_<95% (OR=1,62; IC95% 1,38 - 1,78). Entre os achados radiológicos, destacaram-se infiltrado intersticial (OR=1,65; IC95%1,16 - 2,36), consolidação (OR=2,85; IC95%1,80 - 4,54), misto (OR=2,26; IC95%1,42 - 3,61), outros achados (OR=1,80; IC95%1,23 - 2,63) e não realização do exame (OR=1,52; IC95%1,08 - 2,14). Além disso, o aspecto da tomografia indeterminado para COVID-19 (OR=1,40; IC95% 1,01 - 1,93) e a não realização da tomografia (OR=0,53; IC95% 0,32 - 0,89) também se destacaram, assim como os resultados da imunofluorescência (OR=1,66; IC95%1,25 - 2,21). As variáveis como tosse (OR=0,73; IC95% 0,65 - 0,2), dor de garganta (OR= 0,80; IC95% 0,70 - 0,91) e teste antigênico não realizado (OR=0,53; IC95% 0,32 - 0,89) se mostraram como fatores de proteção.

**Tabela 2:** Análise de regressão logística não ajustada e ajustada dos sinais e sintomas, e exames realizados dos casos de óbito em pacientes graves com COVID-19/diabetes *mellitus* no Brasil de 2020 a 2022

Variáveis				ÓBITO		
	n	%	*Odds Ratio* não ajustada (IC95%)	Valor de p^*^	*Odds Ratio* ajustada (IC95%)	Valor de p^*^
Febre						
Sim	100.033	42,1	1,00	-	-	-
Não	58.900	42,6	1,02 (1,00 - 1,03)	0,003	0,96 (0,87 - 1,07)	0,49
Tosse						
Sim	125.522	40,8	0,80 (0,79 - 0,81)	<0,001	0,73 (0,65 - 0,82)	<0,001
Não	40.209	46,2	1,00	-	-	-
Dor de garganta						
Sim	27.310	39,3	0,88 (0,86 - 0,89)	<0,001	0,80 (0,70 - 0,91)	<0,001
Não	105.582	42,4	1,00	-	-	-
Dispneia						
Sim	151.734	45,9	1,81 (1,78 - 1,84)	<0,001	1,38 (1,21 - 1,57)	<0,001
Não	23.020	31,9	1,00	-	-	-
Desconforto respiratório						
Sim	125.182	47,7	1,82 (1,79 - 1,89)	<0,001	1,87 (1,67 - 2,10)	<0,001
Não	36.103	33,4	1,00	-	-	-
SpO_2_<95%	2					
Sim	142.724	46,5	1,86 (1,83 - 1,89)	<0,001	1,62 (1,38 - 1,78)	<0,001
Não	25.998	31,8	1,00	-	-	-
Diarreia						
Sim	21.934	38,5	0,85 (0,83 - 0,86)	<0,001	0,98 (0,85 - 1,14)	0,83
Não	110.320	42,5	1,00	-	-	-
Vômito						
Sim	14.451	39,0	0,88 (0,86 - 0,90)	<0,001	0,93 (0,78 - 1,10)	0,38
Não	115.560	42,2	1,00	-	-	-
Dor abdominal						
Sim	8.660	38,8	1,00	-	-	-
Não	99.738	41,4	1,11 (1,08 - 1,14)	<0,001	1,02 (0,84 - 1,24)	0,82
Fadiga						
Sim	41.830	41,1	1,00	-	-	-
Não	73.827	41,4	1,01 (0,99 - 1,02)	0,137	1,15 (1,03 - 1,29)	0,01
Perda de olfato						
Sim	11.305	33,6	1,00	-	-	-
Não	96.999	42,0	1,43 (1,39 - 1,46)	<0,001	1,13 (0,89 - 1,44)	0,31
Perda de paladar						
Sim	11.491	33,3	1,00	-	-	-
Não	96.779	42,0	1,45 (1,41 - 1,48)	<0,001	1,27 (1,00 - 1,61)	0,054
Raio-X de tórax						
Normal	2.575	34,3	1,00	-	-	-
Infiltrado intersticial	27.078	44,7	1,55 (1,47 - 1,63)	<0,001	1,65 (1,16 - 2,36)	0,005
Consolidação	4.290	53,2	2,17 (2,04 - 2,32)	<0,001	2,85 (1,80 - 4,54)	<0,001
Misto	4.633	50,0	1,91 (1,79 - 2,03)	<0,001	2,26 (1,42 -3,61)	0,001
Outro	12.892	45,0	1,56 (1,48 - 1,64)	<0,001	1,80 (1,23 - 2,63)	0,002
Não realizado	49.870	39,2	1,23 (1,17 - 1,29)	<0,001	1,52 (1,08 - 2,14)	0,02
Tipo de amostra clínica coletada para teste						
Secreção de naso-orofaringe	156.948	42,9	1,00	-	-	-
Lavado broco-alveolar	1.025	65,0	2,47 (2,23 - 2,75)	<0,001	1,81 (0,07 - 42,63)	0,713
Tecido *post-mortem*	77	80,2	5,40 (3,26 - 8,92)	<0,001	0,67 (0,54 - 0,84)	0,001
PCR						
Detectável	133.063	44,1	1,17 (1,14 - 1,19)	<0,001	1,23 (0,98 - 1,56)	0,08
Não detectável	8.642	33,0	0,73 (0,70 - 0,75)	<0,001	0,77 (0,57 - 1,03)	0,08
Inconclusivo	330	39,2	0,95 (0,83 - 1,10)	0,546	1,17 (0,33 - 4,11)	0,80
Não realizado	18.478	44,2	1,17 (1,14 - 1,20)	<0,001	1,05 (0,85 - 1,31)	0,63
Aguardando resultado	17.685	40,3	1,00	-	-	-
Aspecto da tomografia						
Típico COVID-19	65.353	38,6	1,00	-	-	-
Indeterminado COVID-19	3.467	42,6	1,18 (1,12 - 1,23)	<0,001	1,40 (1,01 - 1,93)	0,046
Atípico COVID-19	1.749	40,4	1,07 (1,01 - 1,14)	0,018	0,92 (0,65 - 1,31)	0,64
Negativo para pneumonia	255	26,3	0,56 (0,49 - 0,65)	<0,001	0,40 (0,13 -1,27)	0,12
Não realizado	30.244	47,5	1,44 (1,41 - 1,46)	<0,001	1,20 (1,08 - 1,33)	<0,001
Tipo de teste antigênico						
Imunofluorescência	30.925	46,9	1,14 (1,12 - 1,17)	<0,001	1,66 (1,25 - 2,21)	<0,001
Teste rápido antigênico	33.372	43,5	1,00	-	-	-
Resultado do teste antigênico						
Positivo	36.406	44,0	1,10 (1,09 - 1,12)	<0,001	1,14 (0,70 - 1,84)	0,60
Negativo	2.596	37,8	0,85 (0,81 - 0,90)	<0,001	0,63 (0,38 - 1,05)	0,07
Inconclusivo	17	43,6	1,09 (0,58 - 2,05)	0,785	1,73 (0,26 - 11,43)	0,57
Não realizado	52.442	43,6	1,09 (1,07 - 1,10)	<0,001	0,53 (0,32 - 0,89)	0,02
Aguardando resultado	74.332	41,4	1,00	-	-	-
Teste sorológico (IgM)						
Positivo	13.146	43,5	1,00	-	-	-
Negativo	4.347	47,1	1,15 (1,10 - 1,21)	<0,001	1,20 (0,97 - 1,49)	0,09
Inconclusivo	48	53,3	1,48 (0,97 - 2,24)	0,063	1,54 (0,62 - 3,79)	0,35
Não realizado	11.713	45,5	1,08 (1,04 - 1,12)	<0,001	0,96 (0,83 - 1,10)	0,51
Aguardando resultado	26	44,8	1,05 (0,62 - 1,76)	0,842	1,28 (0,89 - 2,31)	0,08

* *Para validação do modelo, foi realizado o teste de Hosmer-Lemeshow (p=0,11), Nagelkerke (0,29), indicando qualidade e ajuste do modelo; SpO2 - saturação periférica de oxigênio.*

## DISCUSSÃO

O DM aumenta os riscos de complicações fatais na COVID-19^(19)^. Sobre isso, pesquisa conduzida no Brasil evidenciou que três em cada 20 óbitos por COVID-19 foram observados em pessoas com DM, com uma prevalência 1,15 vezes maior entre os casos hospitalizados com DM em comparação aos sem DM^(20)^. Ao analisar a interligação da comorbidade com os fatores sociodemográficos, constatou-se que o risco de mortalidade foi acentuado no sexo masculino. Isso pode estar relacionado aos fatores biológicos específicos, tendo em vista que a expressão quase três vezes maior da Enzima Conversora de Angiotensina 2 nos homens, em comparação com as mulheres, contribui para o agravamento da doença, pois o vírus da COVID-19 utiliza essa enzima como um receptor para adentrar as células^(21,22)^. Além dos aspectos biológicos, barreiras culturais como a construção da masculinidade desempenham um papel significativo na resistência dos homens à busca por serviços de saúde e menor adesão a práticas preventivas, o que torna essa população mais vulnerável ao adoecimento^(23,24)^. Esses fatores exemplificam como os determinantes estruturais e intermediários, como gênero, padrões culturais e práticas de saúde, influenciam o risco e o desfecho da COVID-19 em pessoas com DM.

No que se refere à faixa etária, o aumento do risco de mortalidade esteve presente em adultos e idosos. A respeito disso, os idosos, especialmente aqueles com condições de saúde pré-existentes, têm risco maior de desenvolver formas graves e morte relacionadas à COVID-19 do que pessoas mais jovens^(25)^. Estudo realizado no Brasil com a população geral constatou que pessoas idosas na faixa etária de 60 a 79 anos apresentaram risco 2,87 vezes maior de mortalidade por COVID-19, enquanto idosos com 80 anos ou mais tiveram risco 7,06 vezes maior em comparação com indivíduos com menos de 59 anos e, quando associadas a comorbidades, a probabilidade de óbito aumentou em 9,44 vezes em relação aos indivíduos sem condições de saúde preexistentes^(26)^.

Essa condição pode ser explicada pela deterioração progressiva do sistema imunológico com o envelhecimento, o que aumenta a vulnerabilidade a doenças infecciosas, tornando o prognóstico desfavorável, se combinada com condições crônicas^(27)^. Vale ressaltar que o aumento do risco de óbito observado na população adulta deste estudo pode estar associado ao fato de que essas pessoas tinham DM, o que pode ter ampliado as chances de mortalidade nessa faixa etária. Dessa forma, a idade, enquanto determinante estrutural, interage com os determinantes intermediários, como a presença de comorbidades, potencializando os desfechos negativos.

Nesta pesquisa, as raças parda, preta e indígena estiveram relacionadas ao aumento no risco de óbitos em pacientes graves com COVID-19/DM. De modo semelhante, estudo realizado no Reino Unido, que avaliou a associação entre raça/cor e sobrevivência em pacientes hospitalizados pela COVID-19, identificou taxas de mortalidade mais elevadas em indivíduos de etnia não branca em comparação com aqueles de etnia branca^(28)^. Estudo brasileiro verificou que a coloração de pele não branca aumentou em 13% o risco de óbito por COVID-19^(26)^.

A predominância de notificações entre indivíduos pardos na amostra resulta da diversidade racial brasileira, que é caracterizada por uma parcela significativa de pessoas que se autodeclaram como pardas^(29)^. Com relação à raça preta, esta enfrenta uma dupla vulnerabilidade, pois além da prevalência de diagnósticos de doenças crônicas, como a DM, essa população também enfrenta desafios ao acesso a serviços básicos, como a saúde, devido a barreiras sociais, como a desigualdade^(30)^. Essa vulnerabilidade à COVID-19 é igualmente evidente entre os povos indígenas, pois enfrentam desafios como escassez de recursos, pobreza e saúde precária. Assim, raça e cor configuram um importante determinante estrutural, associado a condições precárias de vida e de saúde, que amplifica a exposição aos determinantes intermediários e às barreiras no acesso à atenção à saúde.

É relevante destacar que essa desigualdade pode ser compreendida à luz do racismo, que atua como um determinante estrutural ao influenciar o processo de saúde-doença^(14)^. As populações não brancas estão desproporcionalmente representadas entre os grupos com menor renda, menor escolaridade e condições precárias de acesso aos serviços de saúde, o que afeta a transmissão do vírus e aumenta as complicações relacionadas à COVID-19^(31)^.

Neste estudo, diferentes níveis de escolaridade estiveram associados à ocorrência de óbito, contudo a maior chance foi apontada para aqueles sem escolaridade/analfabetos. Sobre isso, estudo brasileiro também identificou a baixa escolaridade como um fator sociodemográfico de risco para o desfecho de morte em casos de SRAG por COVID-19^(32)^. Isso indica que a baixa escolaridade pode representar uma barreira ao acesso à informação, resultando em conhecimento insuficiente sobre a doença, o que pode acarretar dificuldades no controle e no autocuidado, especialmente em pessoas com DM, em que essas dificuldades podem refletir diretamente no controle glicêmico e ocasionar alterações no comportamento relacionado ao autocuidado^(33)^. Nesse contexto, a escolaridade se configura um determinante estrutural fundamental, pois influencia diretamente o acesso à informação, à renda e aos serviços de saúde, além de impactar a capacidade de compreender e adotar práticas de autocuidado que são importantes no manejo de doenças crônicas como o DM. Esses fatores, por sua vez, afetam os determinantes intermediários e contribuem para a ampliação das desigualdades em saúde.

A elevada taxa de óbito associada aos casos nosocomiais corrobora pesquisa realizada na Suécia, na qual a infecção por SARS-CoV-2 adquirida nos hospitais durante as fases iniciais da pandemia resultou em aumento significativo no risco de mortalidade^(34)^. No contexto do DM, a suscetibilidade a infecções hospitalares pode ser explicada pelo comprometimento do sistema imunológico. Essa condição compromete a capacidade do organismo de resistir a qualquer infecção, tornando essas pessoas mais vulneráveis^(8)^. Esse cenário evidencia a complexa interação entre os determinantes intermediários, como a presença de comorbidades, e os fatores relacionados ao tipo de atendimento recebido, como a hospitalização e o suporte em UTI, os quais influenciam diretamente a gravidade do quadro clínico e os desfechos em saúde, como a ocorrência do óbito.

Além disso, doenças cardiovasculares crônicas, asma, doenças renais crônicas e pneumopatias crônicas foram identificadas como fatores de risco para o óbito em pacientes graves com COVID-19/DM. Acerca disso, as DCNT se evidenciam como um problema de saúde global que contribui para o aumento significativo no número de mortes prematuras, destacando-se como prioridade na Agenda de 2030 proposta pela Organização das Nações Unidas (ONU)^(16)^. No Brasil, os óbitos por DCNT aumentaram em 49,6%, totalizando 337.098 mortes em 2019^(35)^. Ademais, a interseção entre DCNT e COVID-19 aumenta a vulnerabilidade desses pacientes, porque o prognóstico de pessoas com comorbidades pré-existentes é desfavorável quando comparado ao de populações fisicamente saudáveis^(36)^. Essas condições constituem determinantes intermediários, pois estão diretamente relacionadas ao estado de saúde dos indivíduos. Quando somadas a outras vulnerabilidades, como dificuldades no acesso aos serviços de saúde ou baixa qualidade do atendimento recebido, tais agravos não transmissíveis potencializam os riscos, por influenciar negativamente o curso clínico e aumentar a probabilidade de óbito em pacientes graves com COVID-19/DM.

Outro aspecto importante a ser mencionado é que, nesta pesquisa, as pessoas com DM que são imunodeficientes ou imunodeprimidos apresentaram risco elevado de mortalidade por SRAG devido à COVID-19. A literatura reforça essa associação, indicando que pacientes com duas ou mais comorbidades possuem mais chances de morrer por COVID-19 do que aqueles com uma única comorbidade ou nenhuma. Além disso, a própria infecção por COVID-19 é conhecida por desregular o sistema imunológico, contribuindo para um pior prognóstico^(37)^.

O presente estudo também evidenciou que pessoas com DM que não foram previamente hospitalizadas/internadas expressaram maiores chances de óbito. Apesar desse resultado, a literatura ressalta que pessoas com DM possuem maior probabilidade de serem hospitalizadas por SRAG devido à COVID-19, por terem um risco aumentado de desenvolverem formas mais graves da doença^(38)^. Esses achados podem ser atribuídos pelo atraso e hesitação na busca por cuidados médicos. Quanto a isso, a disseminação de notícias falsas e imprecisas sobre a doença, gravidade clínica e opções de tratamento não apoiadas por dados clínicos, especialmente no início da pandemia, podem ter gerado confusão e comportamentos de risco^(39)^. Tais achados revelam dificuldades importantes relacionadas ao acesso oportuno e à qualidade da atenção em saúde, que atuam como elementos mediadores entre os determinantes estruturais (como escolaridade, renda e local de residência) e os determinantes intermediários (como presença de comorbidades e sintomas clínicos). A forma como esses fatores são manejados no sistema de saúde pode influenciar diretamente os desfechos clínicos, como a gravidade da doença e o risco de morte.

Constatou-se, ainda, a relação entre a necessidade de suporte ventilatório e internação em UTI e os óbitos em pacientes graves com DM e COVID-19. Isso ocorre porque a internação em UTI e o suporte ventilatório invasivo ou não invasivo são indicativos de um quadro crítico da SRAG^(40)^. Somado a isso, as alterações metabólicas, como a diminuição da resposta imunológica e o aumento de inflamação em pessoas com DM, elevam os riscos de complicações graves e mortalidade por COVID-19^(41)^. Esses fatores representam determinantes intermediários da saúde, como as comorbidades e a gravidade do quadro clínico, que, ao interagirem com elementos do cuidado em saúde, como o tempo de início do tratamento, a qualidade da assistência prestada e a disponibilidade de recursos hospitalares, podem intensificar a vulnerabilidade dos pacientes e impactar negativamente os desfechos clínicos.

Em relação às alterações fisiopatológicas do DM, essa condição não altera apenas a função pulmonar, mas também aumenta a permeabilidade dos vasos sanguíneos e afeta as células epiteliais dos alvéolos. Tal fato pode justificar a maior ocorrência de sintomas respiratórios nos casos fatais em pessoas com DM acometidos por SRAG decorrente da COVID-19, como dispneia, desconforto respiratório e SpO_2_<95%^(42)^. Esses sintomas respiratórios representam determinantes intermediários clínicos, pois refletem a gravidade da condição de saúde do paciente no momento do atendimento. Sua presença está diretamente relacionada a um quadro clínico mais grave, indicando maior comprometimento pulmonar, o que eleva o risco de complicações e óbito.

De modo geral, os pacientes com DM que foram a óbito por quadro grave de COVID-19 realizaram exames sorológicos, incluindo RT-PCR e teste de imunofluorescência para a identificação da presença do vírus. De acordo com os protocolos de diagnóstico da COVID-19, recomenda-se o uso de testes moleculares, como a PCR de Transcrição Reversa Quantitativa, como método de referência para o diagnóstico da doença no Brasil, devido à sua alta precisão e confiabilidade^(4)^. Nesta investigação, os achados radiológicos anormais por raio-X de tórax, aspecto indeterminado para COVID-19 na tomografia e não realização desse exame, apresentaram associação estatisticamente significativa com o desfecho de óbito. Isso reforça a importância dos exames de imagem no diagnóstico da COVID-19, uma vez que a não realização desse tipo de exame compromete, especialmente em situações mais graves, a análise da extensão da doença, diagnósticos alternativos e identificação de complicações^(43)^.

A disponibilidade e a efetiva realização de exames diagnósticos, como a radiografia de tórax e a tomografia, são elementos fundamentais da atenção em saúde, pois influenciam diretamente a precisão do diagnóstico, a definição da gravidade do quadro clínico e a tomada de decisão terapêutica. Quando esses exames não são realizados, especialmente em casos graves com os decorrentes da infecção pelo SARS-CoV-2, há uma limitação na avaliação médica, o que pode atrasar intervenções apropriadas e aumentar o risco de desfechos desfavoráveis, como o óbito.

Importa salientar que, neste estudo, as variáveis que no modelo final de regressão se apresentaram como fator de proteção para o óbito foram residir em zona rural, apresentar tosse, dor de garganta e não ter realizado o teste antigênico. Acredita-se que a associação entre zona rural e menor risco de óbito esteja relacionada à menor densidade populacional; assim, nas zonas rurais, ocorreriam menor transmissão do vírus, diferindo do que ocorre nas regiões metropolitanas^(44)^. Entretanto, a zona de residência, como determinante estrutural, também pode influenciar o acesso aos serviços de saúde especializados, representando tanto fatores protetivos quanto limitantes, dependendo do contexto que está sendo analisado. Apesar de serem pacientes graves, sintomas como tosse e dor de garganta podem atuar como fator de proteção, sugerindo que a infecção ainda esteja restrita às vias aéreas superiores, com uma resposta inflamatória menos sistêmica, o que favorece diagnóstico e tratamento precoces^(45)^. Em relação à não realização do teste antigênico, essa variável pode refletir o acesso a outros métodos de diagnóstico mais sensíveis, favorecendo a condução adequada do caso e melhores prognósticos.

Embora tenham-se identificado associações estatisticamente significativas entre os óbitos de pacientes graves com COVID-19/DM e variáveis como não ser puérpera, não ter doença hepática crônica e doença neurológica, destaca-se que os dados analisados não forneciam informações específicas sobre cada característica associada de maneira isolada. Portanto, é possível considerar que esses pacientes poderiam apresentar outros fatores de risco não contemplados na pesquisa, os quais poderiam contribuir para o desfecho do óbito.

### Limitações do estudo

Como limitação, destaca-se que, por se tratar de estudo com dados secundários, existe a possibilidade da subnotificação dos dados e preenchimento incorreto pelo profissional executante da notificação. Outro fator importante é a opção “ignorado” disponível nas variáveis preenchidas na ficha, o que impossibilita a interpretação minuciosa dos dados.

### Contribuições para as áreas da enfermagem, saúde ou políticas públicas

Os estudos epidemiológicos se mostram importantes, pois fornecem a base científica necessária para a adaptação das práticas de enfermagem, influenciando diretamente a formulação de políticas de saúde pública voltadas ao controle da doença e monitoramento da sua evolução, especialmente diante de desafios emergentes. Além disso, colaboram diretamente para o alcance de metas definidas pela Agenda de 2030 ao alinhar essas práticas ao ODS “Saúde e Bem-estar” da ONU, na promoção de um futuro mais saudável. Ao compreender esses padrões epidemiológicos associados ao DM, os profissionais de enfermagem podem aprimorar habilidades de prevenção, controle e tratamento direcionadas a essa população, visando à redução da prevalência do DM e melhora da qualidade de vida dos pacientes, juntamente com o planejamento e implementação de ações para futuras crises sanitárias.

## CONCLUSÕES

Com base no estudo, foi possível verificar que fatores sociodemográficos, clínicos e de atendimento estiveram associados ao óbito em pacientes graves com COVID-19/DM como: sexo masculino; faixa de 19 a 59 anos; idosos; raça/cor não branca; baixa escolaridade; casos nosocomiais; internação em UTI; suporte ventilatório; doenças cardiovasculares crônicas; asma; pneumopatias crônicas; imunodepressão e doenças renais crônicas; desconforto respiratório; dispneia; saturação <95%; exames de RT-PCR; imunofluorescência; aspecto da tomografia indeterminado para COVID-19; atípico para COVID-19; negativo para pneumonia; e não realizado. A variável zona rural apresentou-se como fator de proteção. Nesse sentido, destaca-se a complexidade dessa comorbidade, enfatizando a importância de intervenções direcionadas a grupos de maior vulnerabilidade e a necessidade de estratégias efetivas de prevenção, monitoramento e tratamento para reduzir o impacto fatal dessa interação.

Esses resultados refletem a inter-relação complexa entre os determinantes estruturais e intermediários da saúde, conforme o modelo dos DSS. Neste contexto, as intervenções direcionadas a grupos mais vulneráveis, com base nos DSS, são fundamentais para reduzir as desigualdades no acesso à saúde e promover a equidade no tratamento. Estratégias de prevenção, monitoramento e tratamento eficazes devem ser desenhadas levando em consideração esses determinantes, a fim de mitigar o impacto fatal da interação entre DM e COVID-19, especialmente em populações em situação de maior vulnerabilidade social e de saúde.

## Data Availability

Os dados de pesquisa estão disponíveis em repositório: https://doi.org/10.48331/scielodata.0RJPTY.
